# Artificial Intelligence (AI) in the Financial Sector—Potential and Public Strategies

**DOI:** 10.3389/frai.2019.00016

**Published:** 2019-10-04

**Authors:** Stephan Bredt

**Affiliations:** Ministry of Economics, Energy, Transport and Housing of the State of Hessen, Wiesbaden, Germany

**Keywords:** artificial intelligence, public strategies, financial sector, national strategy for artificial intelligence, artificial intelligence and market surveillance, artificial intelligence ecosystem frankfurt rhein main, start up ecosystem frankfurt rhein main

## Abstract

AI is providing a significant basis for future technological innovation. The financial sector will be transformed by AI, offering the opportunity for better and more tailor-made services, cost reduction, and the development of new business models. The Federal and the Hessen governments recently published roadmaps for the further development of AI in Germany and Hessen, respectively. The Federal Government will invest three billion euros over the next 5 years in a variety of research and business sectors whereas the State of Hessen will set up a new AI-oriented institute of applied research and business development and spend one billion euros over the next 5 years on digitalization development. The public strategies for building AI hubs are still extremely diverse. However, the focus is on stronger application of research results in business activities, on increasing networks and ecosystems and predominantly on building on existing centers of excellence. The Frankfurt Rhein Main Region, already a strong hub for fintech, cyber security, and AI, will especially benefit from these programs. The financial center Frankfurt offers a vivid and fast growing tech and start up community a well as an academic and data infrastructure unprecedented in Europe: the largest data and cloud service hub in continental Europe, the worlds largest internet knot, universities, research institutes with global quality research in AI, as well as companies and consultancies specialized in AI and neighboring areas such as fintech and cyber security.

## Increasing Importance of AI for Societies and the Economy

AI is recognized as a combination of new technologies, processes, and methods with an increasing importance for the current and future development of our societies and economies. AI is applied today in various diverse sectors such as medical diagnostics, optical character recognition, automotive autonomous driving, and financial services. Already today large corporates and small and medium enterprises in Germany use AI technologies. AI has become a part of daily life for millions of consumers. The application of AI is seen as a potential driver of disruptive technological development and innovation.

That is why recently a flood of studies and reports by research and public institutions as well as consultancies has been published to clarify the opportunities, challenges, and steps ahead (Schwab K., World Economic Forum, 2016, The fourth industrial revolution: what it means, how to respond, 2016; Bafin, Big Data trifft auf künstliche Intelligenz, Herausforderungen und Implikationen für Aufsicht und Regulierung von Finanzdienstleistungen, 2018; BMBF, Die Revolution der Künstlichen Intelligenz, 2018).

Estimates calculate that productivity in Germany could be increased by 29% until 2035 (Accenture, Why AI is the Future of Growth, 2016). Investment in AI is increasing sharply, in start ups as well in existing companies. It is calculated that in 2017, about 17 billion euros were invested globally in AI technology (Accenture, Weg ohne Ziel? Wie Deutschland ein Spitzenstandort für Künstliche Intelligenz werden kann, 2018). Additionally AI will have an increasing impact on financial services. It is estimated that by 2035, banks could improve their productivity annually by 4.3%^1^. In financial services business, AI could transform the financial sector in the following three aspects.

First, AI could improve the quality of products and service for clients due to a broader and deeper analytical basis and information. Second, AI could lead to higher efficiency and lower costs, e.g., in the area of compliance and fraud detection or anti-money laundering measures. Furthermore, public institutions like Financial Market or Tax Supervisory authorities could benefit from AI technology in that sense (Bafin, Big Data trifft auf künstliche Intelligenz, Herausforderungen und Implikationen für Aufsicht und Regulierung von Finanzdienstleistungen, 2018). Third, AI could become a central innovation driver. Although it is not quite clear yet what the financial service provider of tomorrow will look like, it seems probable that AI will transform financial service providers into data- and AI-based businesses (Accenture, Hessens Ambitionen für Künstliche Intelligenz, 2018, p. 11s.).

In Germany, the Federal Government and the State of Hessen government have set up strategic road maps to develop AI made in Germany and AI made in Hessen. These roadmaps are first instruments to shape the development of AI in the financial sector and beyond. They must be embedded in broader public strategies such as development of research and the creation of an innovative environment for start ups and incumbents.

## Strengthening the Tech and AI Ecosystem

The Hessen Government has analyzed the situation of the Frankfurt Metropolitan Region for its status and prospects as a Tech and AI Hub in recent months and years and developed a road map, and it has taken steps to develop it along this roadmap. After the set up of the TechQuartier in 2016, a Masterplan for the region as a Start Up hub was developed in 2017 (Techquartier, Masterplan Start-Up Region Rhein Main, 2018). Subsequently strengths and opportunities of the region were analyzed by the Frankfurt ecosystem report by Startup Genome in 2018. Finally, an analysis by Accenture on the status and opportunities of the region in the AI sector with concrete proposals was presented in 2018. These reports are the basis of the following section.

### The Environment: The Tech Ecosystem in Frankfurt Metropolitan Region

Alongside the public universities with more than 100,000 students, more than 20 private universities and other institutions of higher research, Frankfurt boasts world-class research organizations such as the Fraunhofer Institutes, Leibniz Association, and Max Planck Institutes, among others. The region's 22 research institutions are responsible for breakthrough research, innovative products, and new processes. Academic research in Frankfurt is complemented by corporate research and development (R&D) in production, life sciences, robotics, and artificial intelligence. Corporate R&D spending reached 5.5 billion euros in 2017 (Startup Genome, Frankfurt Startup Ecosystem Report, 2018, p. 16).

The Frankfurt Metropolitan Tech ecosystem can be characterized by three sub-sectors for which the region has potential to build global competitiveness and economic value. These are closely integrated, with alignment across talent and the types of problems that startups are addressing: 1. Fintech, 2. AI, Big Data & Analytics, and 3. Cybersecurity.

Whereas Fintech is characterized in the region by its dynamic start up environment, the exceptional role of the region in cyber security is shaped by research institutions. The Center for Research in Security and Privacy (CRISP) in Darmstadt alone is host to more than 450 researchers in this sector and is complemented by research at the universities and the Fraunhofer Institute. Recently, Chancellor Merkel announced that these institutions will be supported by the Federal Government and be developed as the national cyber security hub.

The creation of the TechQuartier by the State of Hessen with the support of numerous financial service providers in 2016 provided a fast growing platform an light house and most important ecosystem for start ups, businesses, and researchers. The TechQuartier (TQ) is the central platform for the startup community in the Frankfurt Metropolitan Region, enriching the vibrant startup ecosystem with its unique community of more than 100 start ups and 30 corporate partners and academic institutions. Nearly 400 tech start ups are now active in the Frankfurt region, and recent exits and funding rounds have helped drive total ecosystem value to $1.8 billion. These start ups enjoy a supportive environment that includes 32 incubators, 24 coworking spaces, and 10 accelerators.

In 2018, the so called Masterplan Start-Up Region Frankfurt Rhein-Main, developed by TQ and partners was presented. It endorses an ambitious strategy to develop Frankfurt Metropolitan Region as the leading FinTech-Hub in continental Europe and home to 1,000 start ups in 2022.

Frankfurt's multinational corporations operate several programs to support start ups and, increasingly, these companies are investing more into early-stage companies. This has helped Frankfurt achieve one of the fastest growth rates in early-stage funding in the world in recent years. As a center of global finance—home to the European Central Bank and several international banking headquarters—this corporate support has led to one of the world's strongest clusters of Fintech startups. Frankfurt's leading Fintech sub-sector has been catalyzed by financial support (over half of venture capital investment in the region has gone into Fintech startups in the last 5 years) and the $800 million acquisition of 360T in 2015 by Deutsche Börse (See Startup Genome, Frankfurt Startup Ecosystem Report, 2018, Startup Genome, p. 5). However, there are still challenges to the Frankfurt Start Up Ecosystem:

- Over the past few years, Frankfurt has enjoyed one of the world's fastest growth rates in early-stage funding. Yet as startup output rises, that growth needs to continue and even increase, meaning that Frankfurt will need millions of additional dollars per year in early-stage funding (European Parliament, 2018, p. 6).
- Frankfurt has several areas of latent entrepreneurial talent that need to be better activated (European Parliament, 2018, p. 6).
- Frankfurt currently faces gaps in scaleup creation. The exception is the extent to which founders in Frankfurt report the ways they build Global Connectedness (such as meeting founders from international ecosystems locally). In January 2019, TQ, Goethe University and Yi Shi Foundation published a report that addresses this problem and indicates which instruments and strategies could help to overcome this problem (TechQuartier, Goethe University and Yi Shi Foundation for innovations, Scale-Ups In Europe, an untapped potential, 2019).
- The gaps in Frankfurt's startup ecosystem relate to the global orientation of the ecosystem and experience levels (Ecosystem Report p. 39).

### The AI Ecosystem

The AI research in the Frankfurt Metropolitan region has been classified as competitive on a global level (Accenture, Hessens Ambitionen für Künstliche Intelligenz, 2018, p. 15).

The Technical University Darmstadt belongs to the most important universities globally in informatics. Research at TU Darmstadt covers the full scale of AI research (Machine Learning, Reinforcement Learning, Deep Learning, Supervised und Unsupervised Learning, Computer Vision, NLP, Robotik, Predicative Systems). The Technical University Darmstadt runs an Autonomous Systems Lab for Machine Learning for Intelligent Systems and Robotics with research centered around the goal of bringing advanced motor skills to robotics using techniques from machine learning and control (European Parliament, 2018, p. 22).

Additionally the Frankfurt Big Data Lab Start-up Program at Goethe University offers general training courses for data computation and analytics by startups. Frankfurt School of Finance & Management runs a Center for Human and Machine Intelligence (HMI), which conducts basic and applied research at the intersection of artificial intelligence and machine learning, decision and social science, and finance and management. Just recently, January 2019, the Frankfurt School opened their AI Lab as a place for testing, learning, and developing AI-based ideas and strategies.

Also on the business and start up side, there are growing activities: Some 8.5% of all startups in Frankfurt are in the Artificial Intelligence or Big Data & Analytics sub-sector and, over the past 5 years, the sub-sector captured 13% of all local VC investment (European Parliament, 2018 p. 22).

Still, there are challenges that need to be addressed: more AI talents need to be educated. There are not enough and additionally they do rarely move to finance professionally (Accenture, Hessens Ambitionen für Künstliche Intelligenz, 2018). The number of students in informatics in Hessen increased in the last 10 years by ca. 73% to 1,897 in 2016/2017. However, still only 0.8% of all students in Hessen study informatics (without mixed curricula such as “Wirtschaftsinformatik”). The top employers of graduates from German universities in informatics are Google (25.2%), BMW Group (10.6%), Microsoft (10.5%), Apple (9.9%), and SAP (9.7%). Deutsche Bank is the first financial service provider in that list and is ranked 53 (1.3%).

## Public Strategies for Supporting AI in Germany and Hessen

### The National AI Strategic Report

Germany's national government published its AI Strategic Report in December 2018. The strategy is broad in both focus of industries and technologies as well as in instruments to strengthen AI in Germany (Bundesregierung, Strategie Künstliche Intelligenz der Bundesregierung, 2018). The financial sector is included beside many other business areas (Bundesregierung, p. 25). The instruments mentioned are strengthening the startup environment in AI, building on existing instruments like the Digital Hub Initiative of the Federal Government, the creation of new institutions like the Agency for Disruptive Technologies, 12 centers and “application hubs,” the expansion of venture capital offering, extended research (100 professor positions), and the creation of academic networks (Bundesregierung, p. 6). Industry-supported or -led initiatives can also be eligible for support.

The program will support institutions nationwide that already are focused on AI technology such as the Deutsches Zentrum für Künstliche Intelligenz or Fraunhofer Institutes and specialized universities. The federal government will cooperate closely with the federal states for an effective execution of the program.

The State of Hessen and especially the Frankfurt Metropolitan Region will potentially benefit from that national program. The TechQuartier and the Digital Hub in Darmstadt are both included in the Federal Digital Hub Strategy and are therefore potentially eligible for financial assistance. Also, the abovementioned inclusion of the financial sector and its supervisory institutions offers widespread opportunities for projects to be supported by the national program. The Darmstadt Digital Hub, also part of the Federal Digital Hub Strategy and focused on cyber security, could also benefit from the program. Besides, there are points of contact with national programs that are already enrolled in the Frankfurt Metropolitan Region such as cyber security and so called “Mittelstandsförderung for Digitalization.”

It needs to be decided in the future where exactly some of the three billion euros to be spent by the federal government until 2015 will be invested. The idea exposed in the national program is that this support will be leveraged by investment of academic, commercial, or other public institutions such as the federal states (Bundesregierung, p. 6).

The program has been welcomed and criticized. Welcomed, because it offers for the first time a more comprehensive view and action plan to this important topic, and because it is including concrete approaches and resources. It was criticized because it is missing a kind of focal point but is comprising potentially too many institutions and topics and therefore losing traction and visibility. However, with its decentralized approach, it can be stated that the strategy paper corresponds very well with the German academic and economic national institutional set up. It is left to the competition and efficiency of several academic and economic institutions and players where lighthouses of AI will develop. Still, it would be an advantage and should be a target to develop a globally renowned top institution for AI. Fort the moment the German Center for AI in Saarbrücken and Kaiserlautern is seen as such an institute, which will also benefit from the national strategy. The national strategy could potentially and adequately strengthen this and other players.

### The State of Hessen AI and Tech Strategy

Parallel to the creation of the national strategy for AI Made in Germany, the Hessen State government decided to build up an AI hub in the Frankfurt Metropolitan Region in August 2018. This decision was supported by an analysis of the AI capacities of the Frankfurt Metropolitan Region by Accenture for the State of Hessen (Accenture, Hessens Ambitionen für Künstliche Intelligenz, 2018). Before, an analysis from Startup Genome had already encouraged increasing the support for the development of AI start ups in the Frankfurt Metropolitan Region (European Parliament, 2018). The core finding and then proposal of the Accenture report was that there are already high quality research and business activities in the region but that sufficient interconnectedness was missing. Also, a finding was that research results should be more effectively introduced into business activities.

These findings and corresponding proposals and measures were then inshrined in the Coalition Treaty 2018 in Hessen. A specific focus in the coalition treaty is on the development of AI (Koalitionsvertrag zwischen CDU Hessen und Bündnis90/Die Grünen Hessen für die 20. Legislaturperiode, 2018 p. 178s). It includes the creation of a tech campus with 20 professor positions and is supposed to overcome the shortcomings found by the Accenture analysis concerning interconnectedness in the AI ecosystem in the Frankfurt Metropolitan Region.

The tech campus is supposed to strengthen applied research in AI and deliver a growing number of coders and IT specialists for a growing AI economy. It seems open to decision currently, which kind of institution the TechCampus will develop into. There exist several successful tech campuses in Germany which could serve as a role model: the CDTM in Munich, the Code University in Berlin, and the Hasso Plattner Institute at Potsdam University. Other federal states and cities in germany have also published plans to develop such campuses, e.g., the states of Hamburg and North Rhine Westphalia.

Already now, AI activities have been intensified by activities of TechQuartier and industry partners concerning the ecosystem, comprising accelerator programs and seminars. The next step under preparation by the Ministry of Economics together with TQ and industry partner is to make use of the outstanding data infrastructure the region provides: national and Europe-wide data are available with the federal statistics office in Wiesbaden, the Bundesbank research center in Frankfurt, the Goethe University participating in a Europe wide data project on financial data going back to the nineteenth century, the ECB, and EIOPA und Bafin collecting financial data broadly and in depth. Besides, Frankfurt is home to the continent's largest offers in cloud services and data centers, of commercial financial data providers such as Deutsche Börse and Schufa. It will be an opportunity and challenge to make use of these data pools for AI purposes. The idea is to set up or open platforms as far as possible for start ups and new technologies and applied research as the provision of sufficient data is understood as the most relevant basis of AI applications. There will be public and private interest in projects to be developed on these platforms: financial market supervision instruments for the supervisory mentioned above and based in Frankfurt or AI-based tools for business processes in diverse industries. Moreover, university labs could offer access for students to such data pools.

Legal restrictions in the EU and Germany are simultaneously both chance and impediment: chance, because a safe and reliant legal environment attracts data providers and companies outsourcing data, and impediment because a broad use of these data is still often prohibited; some may only be used for academic research, others may not be combined with other specific data. In general, it will be a challenge to define broad limits for the outsourcing for companies' data. The security of data stored in cloud service could be improved and enhanced for this purpose. An international cloud provider could potentially offer security standards to be fully controlled by the outsourcing company.

The coalition treaty includes also the decision to strengthen the access to venture capital for AI start ups (Koalitionsvertrag, p. 175). It was decided to set up a specialized fund with a volume with up to 200 million euros contributed to publicly and privately equally. It was also decided to invest generally 1 billion euros for “digitalization” measures and programs, concerning public institutions, infrastructure and business development. Besides, the existing structures such as the TQ are being focused more on AI-related projects and startup programs.

## Conclusion

In 2018, public strategies and programs for the development of AI have leaped forward significantly (Basel Committee on Banking Supervision (BCBS), 2018; European Commission, 2018; European Parliament, 2018). Germany and the State of Hessen are investing significant resources to strengthen their already highly competitive AI ecosystems, research, and technology. Other federal states have already set up dedicated technology innovation hubs or are currently planning to do so. The federal AI program will strengthen cooperation of national and state programs and hub development, building on existing centers of excellence. Several analyses have found that the startup ecosystem in Frankfurt Metropolitan Region is fast developing as an early stage ecosystem, and is offering a high potential for development in AI. After the path has been laid with the national and the Hessen AI strategies, the years to come require now efficient execution of these plans and programs.

## Author Contributions

The author confirms being the sole contributor of this work and approved it for publication.

### Conflict of Interest

The author declares that the research was conducted in the absence of any commercial or financial relationships that could be construed as a potential conflict of interest.

## Footnotes

^1^Accenture, How AI Boosts Industry Profits an Innovation, 2017. Available online at: https://www.accenture.com/fr-fr/_acnmedia/36dc7f76eab444cab6a7f44017cc3997.pdf
